# Host genetic variants associated with susceptibility and severity of pneumococcal pneumonia in adult patients

**DOI:** 10.1186/s41479-023-00120-w

**Published:** 2023-12-25

**Authors:** Lucía Boix-Palop, María J. Arranz, Anna Sangil, Beatriz Dietl, Mariona Xercavins, Josefa Pérez, Esther Calbo

**Affiliations:** 1 Infectious Diseases Department, Hospital Universitari Mútua de Terrassa, Barcelona, Spain; 2 Universitat Internacional de Catalunya, Barcelona, Spain; 3 Universitat de Barcelona, Barcelona, Spain; 4 Fundació Docència i Recerca Mútua Terrassa, Barcelona, Spain; 5 Internal Medicine Department, Hospital Universitari Mútua de Terrassa, Barcelona, Spain; 6 Microbiology Department, CatLab, Barcelona, Spain

**Keywords:** Genetic variants, Polymorphisms, Pneumococcal community-acquired pneumonia, Host susceptibility, Severity and outcome

## Abstract

**Background:**

Pneumococcal community-acquired pneumonia (P-CAP) is a major cause of morbidity and hospitalization. Several host genetics factors influencing risk of pneumococcal disease have been identified, with less information about its association with P-CAP. The aim of the study was to assess the influence of single nucleotide polymorphisms (SNP) within key genes involved in the innate immune response on the susceptibility to P-CAP and to study whether these polymorphic variants were associated with the severity and outcome of the episodes in a cohort of adult Caucasian patients.

**Methods:**

Seventeen SNPs from 7 genes (IL-R1, IL-4, IL-10, IL-12B, NFKBIA, NFKBIE, NFKBIZ) were analyzed. For susceptibility, a case-control study including a cohort of 57 adult with P-CAP, and 280 ethnically matched controls was performed. Genetic influence on clinical severity and outcome was evaluated in a prospective observational study including all consecutive adult P-CAP patients from November 2015 to May 2017.

**Results:**

The NFKBIA polymorphism rs696 and a haplotype combination were associated with susceptibility to P-CAP (OR = 0.62, *p* = 0.005 and OR = 0.63, *p* = 0.008, respectively). The SNP IL4 rs2227284 was associated with severe P-CAP (OR = 2.17, *p* = 0.04). IL-R1 (rs3917267) and IL-10 (rs3024509) variants were related with respiratory failure (OR = 3.31, *p* = 0.001 and OR = 0.18, *p* = 0.003, respectively) as well as several haplotype combinations in NFKBIA, NFKBIZ, IL-R1 and IL-10 (*p* = 0,02, *p* = 0,01, *p* = 0,001, *p* = 0,03, respectively). CURB-65 values were associated with the IL-10 rs3024509 variant (beta = − 0.4, *p* = 0.04), and with haplotype combinations of NFKBIZ and IL-10 (*p* = 0.05, *p* = 0.04, respectively). Genetic variants in IL-10 (rs3024509) and in IL-12B (rs730691) were associated with PSI values (beta = − 0.54, *p* = 0.01, and beta = − 0.28, *p* = 0.04, respectively), as were allelic combinations in IL-R1 (*p* = 0.02) and IL-10 (*p* = 0.01). Finally, several polymorphisms in the IL-R1 gene (rs13020778, rs2160227, & rs3917267) were associated with the time elapsed until clinical stability (beta = − 0.83, *p* = 0.03; beta = − 1, *p* = 0.02 and beta = 1.07, *p* = 0.008, respectively).

**Conclusions:**

A genetic variant in NFKBIA was associated with susceptibility to P-CAP in adult Caucasian patients and genetic variants from key cytokines of the innate immune response (Il-4, IL-10, IL-R1 and IL-12B) and NF-κB inhibitors were associated with different phenotypes of severe P-CAP. If validated, these SNPs may help to identify people at risk of P-CAP or severe P-CAP on which preventive measures could be applied.

**Supplementary Information:**

The online version contains supplementary material available at 10.1186/s41479-023-00120-w.

## Introduction

Community-acquired pneumonia (CAP) is a leading cause of hospitalization and mortality worldwide [1, 2]. Before SARS-Cov-2 pandemics, *Streptococcus pneumoniae* was the major CAP etiological agent and was responsible for a significant proportion of CAP mortality [3–5] The COVID-19 pandemic and subsequent lockdown measures have been associated with a large decline in invasive pneumococcal disease (IPD) [6, 7]. Nevertheless, IPD seems to be reaching values similar to the pre-pandemic period during the last year [8].

The risk of developing pneumococcal pneumonia (P-CAP) and the severity of the episodes are conditioned by the interplay between the host susceptibility, the virulence of the pathogen and the appropriateness of antimicrobial treatment, which will determine the characteristics and the magnitude of the inflammatory response [9–11]. Regarding host susceptibility, it is well known that age, comorbidities and vaccination status are related with the development and evolution of P-CAP [12–15]. Recently, it has also been suggested that the lung microbiota could play a role in P-CAP susceptibility and the intensity of the inflammatory response [9, 16]. However, it is still unknown why only a minority of the patients with nasopharyngeal pneumococcal colonization develop P-CAP, as the pathogenesis of pneumococcal pneumonia and its severity is not fully explained by these elements.

Genetic variability affecting the host immune response may also influence the susceptibility, the severity and outcome of P-CAP. At the end of the twentieth century, genetic factors were found to be a major determinant of susceptibility to infectious diseases, according to studies carried out in twins and adoptees [17, 18]. Genetic susceptibility to pneumococcal disease was first identified in extreme-phenotype studies in patients with recurrent invasive pneumococcal disease (IPD) [19]. During the last 20 years single nucleotide polymorphisms (SNP) of several genes encoding for key molecules in pathways of the innate and adaptive host immunity that potentially influence the susceptibility, the severity and outcome of pneumococcal disease have been identified.

Genetic variants of different cytokines of the innate immune response, such as IL-1b, IL1-R1, IL-4, IL-10 or IL-12B, have been associated with susceptibility to IPD in children of different ethnic groups [20]. Given the relevance of the nuclear factor κB (NF-κB), its potential association with susceptibility to IPD has been evaluated, reporting associations with an increased risk of IPD [17]. In a previous study published by our group evaluating the genetic susceptibility to IPD in as cohort of Caucasian adults, genetic variants in NFKBIA, NFKBIE and the genomic distribution of NFKBIZ and IL1-R1 were associated with IPD [21]. A recent meta-analysis showed that variants in CD14 and MBL2 genes were associated with susceptibility to pneumococcal disease, and there were no associations with the severity or outcome [22]. It should be noted that most of the studies performed with genetic variants in CD14 and those that found an association with the MBL2 SNPs were conducted in children and adults with IPD.

Regarding P-CAP, there is less evidence on the association of host genetic variants with the susceptibility and outcome of the episodes. Several polymorphisms in the genes encoding for MBL2, MASP2, SFTPA1, SFTPA2, SFTPD, FCGR2A and FCGR3A have been evaluated [23–29], finding that polymorphisms in SFTPA1, SFTPA2 and SFTPD were associated with susceptibility to P-CAP [26] and gnetic variants in FCGR2 were associated with bacteremic P-CAP. The association between the severity and outcome of P-CAP and the host polymorphisms in TLR2, TLR4, MBL2, MASP2, FCGR2A y FCGR3A, IL-10, TNF-α and IL-6 genes has also been evaluated [23–26, 28–34]. Allelic variants of MBL2 have been associated with mortality in a cohort of patients with sepsis and CAP [31] and with bacteremia [32]. A polymorphism in FCGR2 has been associated with the presence of bacteremia, acute renal failure, severe sepsis and adult respiratory distress syndrome (ARDS) [28]. In relation to cytokines, SNPs of IL-10 have been associated with septic shock [34] and IL-6 genetic variants have been associated with longer survival and lower risk of ARDS, multiorgan dysfunction and bacterial dissemination [30, 33]. However, it remains to be determined the potential association of the genetic variants of NF-κB and of other relevant cytokines in the innate immune response (pro-inflammatory and anti-inflammatory) in relation to susceptibility, severity, and outcome of P-CAP in adults.

The aims of the present study were: (1) to investigate the influence of genetic polymorphisms of seven candidate genes (IL-R1, IL-4, IL-10, IL-12B, NFKBIA, NFKBIE, NFKBIZ) involved in the innate immune response on the susceptibility to pneumococcal CAP (P-CAP) in a cohort of adult Caucasian patients with CAP and (2) to assess whether polymorphic variants of these genes were associated with the severity and outcome of the episodes.

## Methods

### Study design, setting and patients

Two different studies were conducted in Hospital Universitari Mútua Terrassa (HUMT), a 400-bed teaching hospital in Barcelona, Spain; the first to determine the influence of genetic polymorphisms on the susceptibility to P-CAP, and the second to assess their association with the severity and outcome of the episodes.

For susceptibility prediction, a case-control study was performed. Cases were 57 adults with P-CAP from a cohort of 124 patients with CAP prospectively included. Controls were 280 ethnically matched healthy adult blood donors from the same geographic area retrospectively collected from an historic database of our study group [14]. The demographic characteristics of the cases and controls population are summarized in Table 1. Comparisons including P-CAP cases (*N* = 57) and controls (*N* = 280) have ≥85% statistical power to detect genetic associations with moderate effect (d = 0.05, *α* = 0.95, two-sided)(calculated with G*Power v3.1.9.4).

**Table 1:** Characteristics of the case-control population

	Controls (*N* = 280)	Cases: P-CAP patients (*N* = 57)
Age, mean (SD) (years)	60 (17)	65.9 (16.4)
Gender, male (%)	108 (38.6)	39 (68.4)
Ethnicity	Caucasian	Caucasian

Genetic influence on clinical severity and outcome was evaluated in a prospective observational study including all consecutive adult P-CAP patients attending the Emergency Department (ED) from November 2015 to May 2017. The adult P-CAP patients included in this prospective study were also the cases of the case-control study.

CAP was defined as an acute illness (symptoms lasting for ≤7 days) with the presence of a new infiltrate on a chest radiography associated with two or more of the following signs and symptoms: fever or hypothermia, dyspnoea, cough, sputum production, pleuritic chest pain, altered breath sounds on auscultation [3]. The exclusion criteria were: age < 18 years, nosocomial or bronchoaspiratory pneumonia, antibiotic treatment started at the ED > 4 h before the potential inclusion in the study, hospital admission during the previous 14 days or previous inclusion in the study. Only Caucasian subjects were analyzed to reduce genetic heterogeneity.

The study was conducted in accordance with the latest version of the Declaration of Helsinki and was approved by the hospital Ethics and Research Committee (record 11/15). Written informed consent obtained within 72 hours after admission was required for data collection [35].

### Definitions

Pneumococcal CAP (P-CAP) was defined as described in a recently published article by the group [36] (Supplementary Material S1). Invasive pneumococcal disease (IPD) was defined as the presence of *S. pneumoniae* in blood cultures, pleural fluid or cerebrospinal fluid. The pneumonia severity index (PSI) [37] and the CURB score [38] were used to grade the severity of the CAP. The presence of respiratory failure was described as the presence of a PaO2/FiO2 ratio ≤ 300 or a pulse oximetric saturation (SpO2)/FiO2 ratio ≤ 315 [2]. Severe CAP was defined as the presence of septic shock, need of mechanical ventilation (MV) or Intensive Care Unit (ICU) admission.

### Clinical evaluation at admission and follow up

Demographic and clinical variables were prospectively collected as described in reported in the supplementary material section (Supplementary Material S1). The time from symptoms onset to the ED visit (hours) and the time needed to reach clinical stability (days) were reported.

Before starting antibiotic therapy, conventional and molecular microbiological studies, chemistry and haematological tests, arterial blood gas sampling and chest radiography were performed (Supplementary Material S1).

### Genetic characterisation of the sample

Whole blood samples were obtained from each study participant. DNA was extracted using a commercial kit (SQ Blood DNA Kit II, Omega Bio-Tek, USA) and following the manufacturer’s instructions. Samples were stored at -80C until required for study. Seventeen Tag SNPs (r2 = 0.8, minimum allele frequency ≥ 0.05) from 7 genes coding for proteins involved in the innate immunological response (ILR1, IL4, IL10, IL12B, NFKBIA, NFKBIE, NFKBIZ) were investigated. Genotypic characterisation of the samples was conducted using high-throughput techniques (Sequenom MassARRAY platform and iPLEX Gold reaction assays, CEGEN, Santiago de Compostela, Spain). Quality control analyses revealed ≥99% genotyping success rates for all SNPs and samples. All SNPs except ILR1 rs391726 were found in Hardy-Weinberg equilibrium. As a result, the ILR1 rs391726 was excluded from subsequent analyses.

### Statistical analysis

Regression analyses comparing patients versus controls considering the genotypes, age and gender as predictor variables were performed for each individual SNP and for haplotype combinations within a gene to identify possible susceptibility risk genotypes. Further regression analyses considering response phenotypes (severe CAP, respiratory failure, PSI and CURB-65 scores, time to clinical stability) as dependent variables, and genotype, age, gender and Charlson score as predictor variables were conducted to elucidate associations with severity within the patient sample. All comparisons were conducted using the Plink statistical package (version 1.07-dos, Purcell et al. 2007).

Continuous variables are presented as means and standard deviation (SD) or medians and interquartile range (IQR) and categorical variables using counts and percentages. Univariate analyses were performed with t test or Mann-Whitney test for continuous variables and the Chi-square test or Fisher exact test for categorical variables, as appropriate.

Differences were considered statistically significant at the two-sided *p* < 0.05 level. Statistical analyses were performed using the STATA RELEASE 17 software (StataCorp LP, College Station, TX, USA). Multiple analyses corrections (Bonferroni) were calculated considering the number of polymorphisms investigated (17) and comparisons performed (7) to minimise false positives.

## Results

### Clinical and microbiological features of the study population

A total of 124 patients of Caucasian ethnicity with episodes of CAP and genetic data available were included in the study. An aetiological diagnosis was established in 84 episodes (67.7%). Fifty-seven patients had P-CAP (46%), 11 had another bacterial CAP (8.9%) and 16 had viral CAP (12.9%). Co-infection was present in 39 individuals (31.5%); of these, 29 were P-CAP patients with viral co-infection in 27 cases (16 rhinovirus, 3 influenza, 3 coronavirus, 2 respiratory syncytial virus and 1 metapneumovirus, parainfluenza and adenovirus) and bacterial co-infection in two cases (*Haemophilus influenzae* and *Moraxella catarrhalis*). Patient characteristics and comparison between P-CAP and non- P-CAP episodes are shown in Table 2.

**Table 2:** General characteristics of 124 patients with community-acquired pneumonia (CAP) and comparison between pneumococcal community-acquired pneumonia (P-CAP) episodes and non P-CAP episodes

Variable	CAP *N* = 124, N (%)	P-CAP *N* = 57, N (%)	Non P-CAP *N* = 67, N (%)	*p*
**Demographic data**				
Age (years)*	70.5 (57–81)	68 (53.5–79)	74 (57–83)	0.15
Gender, male	78 (62.9)	39 (68.4)	39 (58.2)	0.24
Current smoker	31 (25)	16 (28.1)	15 (22.4)	0.47
Former smoker	45 (36.3)	22 (38.6)	23 (34.3)	0.62
Alcohol abuse	17 (13.7)	7 (12.3)	10 (14.9)	0.67
BMI, mean (SD)	26.6 (4.76)	25.6 (4.6)	27.1 (4.8)	0.09
Influenza vaccine	50 (40.3)	22 (38.6)	28 (41.8)	0.72
Pneumococcal vaccine	17 (13.7)	7 (12.3)	10 (14.9)	0.67
**Comorbid conditions**				
Charlson score*	1 (0–2)	1 (0–2.5)	1 (0–2)	0.66
Immunosuppression ^1^	5 (4)	3 (5.26)	2 (2.99)	0.66
Prior antibiotic treatment ^2^	19 (15.3)	6 (10.5)	13 (19.4)	0.17
Pre-hospital antibiotic treatment ^3^	18 (14.5)	8 (14)	10 (14.9)	0.89
**Clinical features on presentation**				
Time from symptom onset to ED visit (hours)*	72 (48–120)	48 (24–96)	72 (48–144)	0.01
Fever (≥ 38 °C)	51 (41.1)	28 (50.9)	23 (34.3)	0.1
Dyspnoea	90 (72.6)	39 (68.4)	51 (76.1)	0.34
Tachypnea (≥ 20 rpm)	87 (78.4)	44 (83)	43 (74.1)	0.26
Cough	109 (87.9)	52 (91.2)	57 (85.1)	0.3
Purulent sputum	55 (44.4)	22 (38.6)	33 (49.3)	0.23
Pleuritical chest pain	43 (34.7)	28 (49.1)	15 (22.4)	0.002
Septic shock	20 (16.3)	12 (21.1)	8 (12.2)	0.18
Respiratory failure	73 (59.4)	32 (56.1)	41 (62.1)	0.5
Severe CAP ^4^	23 (18.5)	12 (21.1)	11 (16.4)	0.68
PSI classes*	3 (2–4)	4 (3–4)	3 (2–4)	0.42
CURB-65 score*	1 (1–2)	2 (1–2)	1 (1–2)	0.29
Co-infection	39 (31.5)	29 (50.9)	10 (14,9)	< 0.001
**Evolution and Outcome**				
Time to clinical stability (days)*	2 (1–4)	2 (1–4)	2 (1–3.5)	0.33
ICU admission	11 (8.87)	4 (7.02)	7 (10.5)	0.51
Need for mechanical ventilation	6 (5.84)	1 (1.75)	5 (7.46)	0.28
Length of hospital stay (days)*	6 (3–10)	6 (4.5–9)	6 (2–10)	0.78
In-hospital mortality	1 (0.81)	0	1 (1.49)	1

^1^Immunosuppression: chronic corticosteroid therapy, severe neutropenia, solid or hematopoietic organ transplantation, acquired immunodeficiency syndrome and use of chemotherapy, immunosuppressive agents or biological drugs

^2^Prior antibiotic treatment: intake of antibiotic 3 months before hospitalization

^3^Pre-hospital antibiotic treatment: oral intake of antibiotic > 24 hours prior to hospitalization for the same episode of acute disease

^4^ Severe CAP: presence of septic shock, need of mechanical ventilation or Intensive Care Unit admission

*IQR* interquartile range, *SD* standard deviation, *ICU* Intensive Care Unit

* median (IQR)

The genetic studies were performed with the 57 P-CAP episodes. All of them received appropriate antimicrobial treatment, based on beta-lactam monotherapy in 12 episodes (21.1%), quinolones monotherapy in 9 (15.8%), beta-lactam + quinolones in 11 (19.3%) and beta-lactam + macrolide in 25 (43.8%). At admission, severe CAP was present in 12 patients (21.1%) and 32 (56.1%) presented respiratory failure. Thirty-one patients (54.4%) had a high-risk PSI (≥4) and 8 (14%) a high-risk CURB65 (≥3). No patient died during the study.

### Host genetic variants and susceptibility to pneumococcal CAP

The results of the comparisons of the genetic distribution between cases and controls are summarized in Table 3.

**Table 3:** Summary of significant polymorphisms associated with susceptibility to P-CAP in cases compared to healthy controls

Gene	Polymorphism	Reference Allele	Allele frequencies		Statistics	
			*Control*	*Controls*	*OR (95% CI)*	*p*
IL10	rs3024498	G	0.21	0.21	0.95 (0.65–1.41)	0.81
IL10	rs3024509	C	0.07	0.09	1.42 (0.81–2.5)	0.23
IL12B	rs730691	T	0.37	0.39	1.02 (0.74–1.4)	0.92
IL4	rs2227284	A	0.25	0.29	1.12 (0.78–1.59)	0.55
ILR1	rs13020778	C	0.47	0.46	1.02 (0.75–1.38)	0.93
ILR1	rs2160227	A	0.32	0.31	1.03 (0.74–1.44)	0.86
ILR1	rs3917254	A	0.14	0.14	1.03 (0.64–1.65)	0.91
ILR1	rs3917267	A	0.36	0.35	0.92 (0.67–1.27)	0.62
NFKBIA	rs1050851	A	0.19	0.23	1.41 (0.97–2. 05)	0.07
NFKBIA	rs696	A	0.43	0.34	0.61 (0.44–0.85)	0.005
NFKBIE	rs2282151	C	0.19	0.21	1.08 (0.74–1.58)	0.69
NFKBIE	rs730775	G	0.49	0.50	1.09 (0.8–1.5)	0.58
NFKBIZ	rs11718446	A	0.26	0.25	1 (0.69–1.45)	0.99
NKFBIZ	rs645781	C	0.25	0.27	1.07 (0.75–1.55)	0.7
NFKBIZ	rs677011	G	0.36	0.38	1.07 (0.77–1.48)	0.7
NFKBIZ	rs9841857	T	0.27	0.24	0.82 (0.57–1.19)	0.3

*OR* Odds ratio, *CI* confidence interval

A NFKB1A polymorphism, rs696, was significantly associated with susceptibility to CAP (*p* = 0.005) with the rs696-A variant more frequently observed in controls (0.43) than in patients (0.34). Haplotype analyses also revealed a similar association between a NFKB1A allele combination and susceptibility to pneumococcal CAP (*p* = 0.008). No other statistically significant association with susceptibility was observed.

### Host genetic variants and severity of pneumococcal CAP

The results of the statistical analyses on severity data are summarized in Table 4.

**Table 4:** Summary of significant polymorphisms associated with severity and outcome of P-CAP

Gene	Polymorphism	Reference Allele	Severe CAP		Respiratory failure		CURB-65		PSI		Time to clinical stability	
			*OR (CI 95%)*	*p*	*OR (95% CI)*	*p*	*Beta (SE)*	*p*	*Beta (SE)*	*p*	*Beta (SE)*	*p*
IL10	rs3024498	G	1.05 (0.49–2.25)	0.91	0.8 (0.41–1.53)	0.5	0.02 (0.15)	0.91	−0.11 (0.16)	0.51	0.26 (0.45)	0.57
IL10	rs3024509	C	0.17 (0.02–1.39)	0.10	0.18 (0.-0.57)	0.003	−.4 (0.19)	0.04	−.54 (0.21)	0.01	−1 (0.58)	0.09
IL12B	rs730691	T	0.55 (0.27–1.12)	0.10	0.8 (0.46–1.4)	0.43	−.23 (0.13)	0.07	−.28 (0,13)	0.04	−.21 (0.38)	0.59
IL4	rs2227284	A	2.17 (1.02–4.61)	0.04	0.96 (0.52–1.76)	0.89	−.01 (0.14)	0.92	−.07 (0.15)	0.65	−0.02 (0.43)	0.97
ILR1	rs13020778	C	0.84 (0.42–1.67)	0.62	0.64 (0.36–1.14)	0.13	0.06 (0.13)	0.65	0.01 (0.14)	0.94	−0.83 (0.37)	0.03
ILR1	rs2160227	A	0.99 (0.48–2.05)	0.97	0.72 (0.38–1.34)	0.30	0.01 (0.14)	0.93	<.01 (0.15)	1	−1.00 (0.42)	0.02
ILR1	rs3917254	A	0.62 (0.22–1.79)	0.38	0.91 (0.41–2.03)	0.82	−.16 (0.19)	0.40	−.33 (0.2)	0.09	−0.13 (0.55)	0.81
ILR1	rs3917267	A	1.86 (0.9–3.82)	0.09	3.33 (1.62–6.86)	0.001*	0.18 (0.13)	0.19	0.09 (0.14)	0.55	1.07(0.4)	0.008
NFKBIA	rs1050851	A	1.85 (0.9–3.79)	0.09	1.14 (0.58–2.23)	0.71	0.11 (0.14)	0.45	−.10 (0.16)	0.52	−0.19 (0.43)	0.67
NFKBIA	rs696	A	0.88 (0.44–1.78)	0.73	1.55 (0.86–2.8)	0.14	−0.02 (0.13)	0.88	0.15 (0.14)	0.28	0.18 (0.4)	0.65
NFKBIE	rs2282151	C	0.57 (0.24–1.36)	0.21	0.89 (0.47–1.8)	0.71	−.12 (0.15)	0.41	−.12 (0.16)	0.44	0.38 (0.44)	0.39
NFKBIE	rs730775	G	0.79 (0.41–1.53)	0.49	0.75 (0.43–1.29)	0.29	.03 (0.13)	0.84	0 (0.14)	0.99	−0.89 (0.37)	0.02
NFKBIZ	rs11718446	A	1.64 (o.74–3.62)	0.22	0.75 (0.38–1.49)	0.41	0.07 (0.15)	0.64	−.11 (0.17)	0.51	0.08 (0.46)	0.87
NKFBIZ	rs645781	C	0.59 (0.26–1.31)	0.19	0.77 (0.43–1.41)	0.40	−.05 (0.14)	0.74	−.01 (0.15)	0.94	−0.61 (0.41)	0.14
NFKBIZ	rs677011	G	0.79 (0.39–1.6)	0.52	0.82 (0.47–1.43)	0.48	−.11 (0.13)	0.39	0.02 (0.14)	0.89	−0.75 (0.39)	0.06
NFKBIZ	rs9841857	T	1.12 (0.52–2.39)	0.78	1.24 (0.66–2.34)	0.51	0.19 (0.15)	0.19	0.10 (0.16)	0.54	−0.11 (0.44)	0.8

*OR* Odds ratio, *CI* confidence interval, *Beta* beta coefficient, *SE* Standard error

*Adjusted (Bonferroni corrected) *p* value = 0.02

A single nominal association was observed between an IL-4 rs2227284 variant and severe CAP (*p* = 0.04). No other significant single marker or haplotype association with severe CAP was detected. Two polymorphic variants in IL-R1 (rs3917267) and IL-10 (rs3024509) genes were associated with respiratory failure (*p* = 0.001, & *p* = 0.003, respectively) whereas several associations with haplotype combinations in NFKBIA (*p* = 0.02), NFKBIZ (*p* = 0.01), IL-R1 (*p* = 0.001) and IL-10 (*p* = 0.003) genes were also observed. CURB-65 values were marginally associated with the IL-10 rs3024509 variant (*p* = 0.04), and with haplotype combinations of NFKBIZ (*p* = 0.05), and IL-10 (*p* = 0.04). Single markers in IL-10 (rs3024509) and in IL-12B (rs730691) were associated with PSI values (*p* = 0.01& *p* = 0.04, respectively). Haplotype analyses also revealed significant associations between PSI and allelic combinations in IL-R1 (*p* = 0.002) and IL-10 (*p* = 0.01) genes. Finally, several polymorphisms in the IL-R1 gene (rs13020778, rs2160227, & rs3917267) were associated with the time elapsed until clinical stability (*p* = 0.03, 0.02, & 0.008, respectively). Additionally, a single marker in NFKBIE (rs730775) was also associated with time to stability (*p* = 0.02). Marginal associations were also observed between the time to clinical stability and haplotype combinations of IL-R1 (*p* = 0.04) and NFKBIE (*p* = 0.01) variants. It is important to note that none of the reported associations would survive multiple analyses corrections, except for the association between rs3917267 and respiratory failure (*p* = 0.02 after Bonferroni correction, Table 4).

## Discussion

We investigated the influence of 17 SNPs within 7 immunological genes (IL-R1, IL-4, IL-10, IL-12B, NFKBIA, NFKBIE and NFKBIZ) on the susceptibility, severity, and prognosis of P-CAP, in a prospective cohort of adult Caucasian patients with P-CAP and controls matched by ethnicity.

The allele distribution of the NFKBIA polymorphism rs696 and its allele distribution were associated with susceptibility to P-CAP in the case-control study and several associations between SNPs in the IL-4, IL-10, IL-R1, IL-12B and NF-κB inhibitors genes and the different phenotypes of severity and outcome of P-CAP were found.

Regarding P-CAP susceptibility, the NFKBIA polymorphism, rs696, as well as one haplotype combination of the polymorphisms investigated within the gene, were associated with protection for the development of P-CAP. The rs696-A variant was more frequently observed in controls than in patients. No other statistically significant association with susceptibility was observed. In our study, the haplotype combination of the polymorphisms in different NF-κB inhibitors genes (NFKBIA,NFKBIE and NFKBIZ) have been associated with respiratory failure, time to clinical stability and higher punctuations in the CURB-65 score. Previous studies have found relation between NFKBIA polymorphisms and IPD susceptibility, but to our knowledge, the risk of P-CAP in relation to NFKB1A has nit been assessed. In a case-control study conducted in adult Caucasian patients [21] the allelic distribution of a variant (rs1050851) in the NFKBIA gene was associated with IPD susceptibility. Additionally, genetic variants in NFKBIA (rs3138053 and rs2233406) have been associated with protection from IPD in Caucasian patients [39]. Previous population-based case-control studies have also identified associations between susceptibility to IPD in Caucasian and African patients of all ages and common polymorphisms in the genes NFKBIE, NFKBIZ and NFKBIL2, each of which encodes an inhibitor of NF-κB [21, 40, 41]. Although the individual effect sizes are small, the risk variants are common in Europeans, suggesting that these polymorphisms may have a contribution to the burden of IPD in this population [42]. The nuclear factor NF-κB inflammatory pathway plays a key role in the host immune response to pneumococcal infection, and very rare genetic mutations in an NF-κB inhibitor cause immunodeficiency with severe bacterial infection [13, 39]. The genetic control of this major inflammatory pathway is extremely complex and incompletely understood. On one hand, impaired NF-κB activation is associated with immunodeficiency, but there is also evidence that increased levels of NF-κB activation are associated with a worse outcome from sepsis [13, 39].

In the present cohort, the IL-4 polymorphism rs2227284 was associated with the presence of severe CAP. The genotype distribution of the same SNP was found to be associated with IPD susceptibility [21], as well as other genetic variants of the Il-4 gen [22], but there is no previously published evidence on its relationship with severity, neither in patients with IPD nor with P-CAP. IL-4 is a major factor in B cell activation and differentiation and it plays an important role in the pathogenesis of sepsis and inflammation [21]. IL-4 and other cytokines were found associated with sepsis severity, organ failure and death in a cohort of patients with septic shock [43]. However, their exact role in the course of infection is still unknown.

A variant in the IL-10 gene, rs3024509, was associated with protection for respiratory failure and for higher CURB-65 and PSI values in this study. Haplotype combinations in the IL-10 gene were also protective for respiratory failure and highest punctuations in PSI score. Our results are in line with a previous study performed in a cohort of patients with IPD and pneumonia [34], in which an IL-10 polymorphism was also associated with severity, with the development of septic shock due to an enhanced IL-10 release. IL-10 is an anti-inflammatory cytokine that plays an important role in the innate immune response. It is able to counterbalance the potentially harmful effects of the proinflammatory response, playing a central role in tissue resilience and contributing to lung homeostasis [9]. However, the IL-10 excess may induce immunosuppression in bacterial sepsis and increases mortality in P-CAP due to bacterial clearance impairment [34, 44]. IL-10 serum levels have been correlated with the severity and the clinical outcome in sepsis, septic shock and CAP [34] and with a fulminant course of *S. pneumoniae* infection in a murine model of P-CAP [45].

A polymorphic variant in the IL-R1 gene, rs3917267, was associated with respiratory failure and time to clinical stability. Moreover, haplotype combinations in this gene were associated with risk of respiratory failure, time to clinical stability and higher CURB-65 and PSI values. Although previous studies have reported an association between genetic variants of the IL-R1 gene and IPD risk [21, 46], there is no published evidence about their relation with the severity or the prognosis of P-CAP. IL-1 plays a critical role in the host response to pneumococcal infections. Its effects are controlled by two inhibitors, the IL-R1 antagonist is one of them The IL-R1 has anti-inflammatory properties and its deficiency in mice has been related with pneumococcal meningitis, higher mortality and enhanced growth of *S. pneumoniae* [47]. The regulation of IL-1 and IL-R1 is complex with a delicate balance between the beneficial effects of modulating bacterial clearance and the detrimental effects of exacerbating the inflammatory response [46].

In addition, a single polymorphism in IL-12B gene, rs730691, was associated with PSI values in this cohort. No previous associations have been described between the IL-12B gene and the severity of P-CAP episodes. IL-12 is a proinflammatory cytokine that promotes the activation of the cellular immunity by activating T lymphocytes. More studies are needed on the role of the inflammatory pathway of these cytokines to understand the impact of genetic polymorphisms in the severity of P-CAP.

The main limitations of the present study are: (a) The sample size is moderate-low for candidate-gene association studies, although it is a well-characterized cohort of P-CAP patients. (b) The proportion of patients with severe CAP was relatively small and mortality was extremely low, perhaps due to a selection bias. (c) The statistically significant associations found were marginal and did not withstand Bonferroni corrections for multiple analyses (*p* > 0.05) except for one finding. However, Bonferroni corrections are too conservative and consider genetic variants in the same genes as independent events and may lead to false negatives. (d) The functional expression of the genetic associations is not described. Finally, our study was conducted in an specific geographical area including only Caucasian patients, and the results cannot be extrapolated to other settings or ethnicities.

In conclusion, in the present study we describe a novel association between a genetic polymorphism in the NFKBIA gene and susceptibility to P-CAP in adult Caucasian patients and we find evidence that genetic variants from key cytokines of the innate immune response (Il-4, IL-10, IL-R1 and IL-12B) and NF-κB inhibitors are associated with the presence of severe CAP, respiratory failure or higher punctuations in CURB-65 or PSI scores in patients with P-CAP. These findings, if validated, may help to identify people at risk of P-CAP and patients at risk of severe forms of P-CAP on which preventive measures could be applied.

### Supplementary Information

**Additional file 1.**

## Data Availability

All data generated or analysed during the current study are available from the corresponding author on reasonable request.
