# Supplementary material for: Host genetic variants associated with susceptibility and severity of pneumococcal pneumonia in adult patients
Source: Pneumonia (Nathan). 2023 Dec 25;15:18. doi: 10.1186/s41479-023-00120-w (PMC10749500; doi:10.1186/s41479-023-00120-w)
Supplement: Supplementary file 1 — Additional file 1. [file 41479_2023_120_MOESM1_ESM.docx]

**Supplementary material**

*Definitions*

Community-acquired pneumonia (CAP) was defined as an acute illness (symptoms lasting for ≤7 days) with the presence of a new infiltrate on a chest radiography associated with two or more of the following signs and symptoms: fever or hypothermia, dyspnoea, cough, sputum production, pleuritic chest pain, altered breath sounds on auscultation. The exclusion criteria were: age <18 years, nosocomial or bronchoaspiratory pneumonia, antibiotic treatment started at the ED >4h before the potential inclusion in the study, hospital admission during the previous 14 days or previous inclusion in the study.

Given the lack of a diagnostic gold standard, pneumococcal pneumonia (P-CAP) was defined by a composite diagnostic variable: detection of *S. pneumoniae* by conventional methods and/or a positive rtPCR-*lytA* in blood, urine or NP swabs (NP cut-off ≥8000 copies/mL) (1). Defined CM were blood culture, good-quality sputum Gram stain, sputum culture or an immunochromatographic test for detection of *S. pneumoniae* antigen in urine.

Invasive pneumococcal disease (IPD) was defined as the presence of *S. pneumoniae* in blood cultures, pleural fluid or cerebrospinal fluid.

Risk factors for IPD included smoking, COPD or asthma, liver cirrhosis, splenectomy or asplenia, autoimmune disease, hypogammaglobulinemia or hypocomplementemia, active solid or hematologic tumors, immunosuppressive or biological therapies, chronic systemic steroid use (> 20 mg prednisone/day>15 days), neutropenia, and HIV infection.

# - Co-infection: cases in which two different microorganisms were simultaneously identified.

# - Vaccination status: it was assessed from interviews with the patients or their relatives and from reviews of electronic medical records. Patients were considered to be pneumococcal-vaccinated if the 23-valent polysaccharide pneumococcal vaccine had been administered in the five years before admission or the 13-valent conjugate vaccine had been administered previously, and influenza-vaccinated if seasonal influenza vaccine had been administered during the year prior to admission.

- Smoking: patients who had smoked at least 100 cigarettes in their lifetime and still smoked or had quit smoking within the preceding year were classified as current smokers. The rest were classified as former smokers (ever exposed) or not smokers [1].

# - Alcohol abuse: it was considered if alcohol intake was ≥ 3 standard drinks per day.

- Comorbidities: were based on Charlson index [2].

# - Immunosuppression: was defined by the presence of at least one of the following: acquired immunodeficiency syndrome (AIDS), chronic corticosteroid therapy, severe neutropenia, solid or hematopoietic organ transplantation and use of chemotherapy, immunosuppressive agents or biological drugs. Corticosteroid therapy was defined as ≥ 5 mg/day of prednisone or equivalent for at least two previous months.

# - Prior antibiotic treatment: was defined as the intake of antibiotic 3 months before hospitalization.

# - Pre-hospital antibiotic treatment: was defined as the oral intake of antibiotic > 24 hours prior to hospitalization for the same episode of acute disease. Patients were classified as receiving antibiotics if they self-reported prescription or by reviewing the prescriptions from their Primary Care Centre.

- Septic shock: systolic blood pressure of less than 90 mmHg, a MAP < 60, or a reduction in systolic blood pressure of > 40 mm Hg from baseline, despite adequate volume resuscitation, in the absence of other causes for hypotension [4].

- Clinical stability was defined as previously described by Halm et al [5].

# - In-hospital mortality: death to any cause during admission.

*Clinical evaluation and follow up*

All CAP episodes were evaluated by an infectious diseases’ consultant before inclusion. All chest radiographs were reviewed by a radiologist confirming the presence of a new infiltrate.

The following variables were prospectively collected: age, gender, race, vaccination status, immunosuppression (acquired immunodeficiency syndrome, chronic corticosteroid therapy, severe neutropenia, solid or hematopoietic organ transplantation and use of chemotherapy, immunosuppressive agents or biological drugs), clinical, microbiological and laboratory data at admission, evolution, length of stay and outcomes. Variables related to comorbidities (based on Charlson severity index), smoking habit and alcohol abuse were also recorded. Prior antibiotic exposure (in the last 3 months), or acute exposure (last 24 hours before admission) were recorded.

*Microbiological tests*

Conventional methods included sputum, nasopharyngeal (NP) swab (Deltaswab amies, Deltalab, Rubí, Spain) and blood culture. Sputum samples were processed for Gram stain. Sputum samples were considered of good-quality if they had <10 squamous cells and >25 leukocytes per low-power field. Blood cultures were processed with the BacT-Alert® system (bioMérieux, Marcy-Etoile, France). Identification of isolates were performed by MALDI-TOF (matrix-assisted laser desorption/ionization time-of-flight) mass spectrometry and antimicrobial susceptibility was tested by the microdilution method, following the European Committee on Antimicrobial Susceptibility Testing methods and criteria (EUCAST). Immunocromatopraphic test for *Streptococcus pneumoniae* and *Legionella pneumophila* serogroup I antigen detection in urine was performed (BinaxNOW® assays, Abbott).

Multiplex-PCR for respiratory viruses detention was performed in NP swabs (AllplexTM Respiratory Panel 1, 2 and 3, Seegene®, Seoul, Korean Republic): influenza A and B virus; respiratory syncytial virus (RSV) A and B; parainfluenza virus (PIV) 1, 2, 3 and 4; coronavirus 229, NL63E and OC43; human rhinovirus (HRV); adenovirus (AdV); metapneumovirus (MPV); bocavirus (HBoV) 1/2/3/4; and enterovirus (HEV).

In patients with no pneumococcal-CAP, serologic methods (on admission and 3–4 weeks thereafter) were used to detect antibodies against *L. pneumophila, Mycoplasma pneumoniae, Chlamydophila pneumoniae* and *Coxiella burnetii*.

*Statistical analysis*

The variables chosen for inclusion in the multivariate analyses were selected by clinical criteria and those with a univariate *p* value <0.05. A best subset regression procedure was used to identify the most suitable and parsimonious multivariate model based on the Akaike information criterion [2].

*References*

1. Sidhu M, Wong M, Karstaedt A, Jansen KU, Albrich WC, Mareletsi T, et al. Use of a Rapid Test of Pneumococcal Colonization Density to Diagnose Pneumococcal Pneumonia. Clin Infect Dis. 2011;54(5):601–9.

2. Akaike H. A new look at the statistical model identification. IEEE Trans. Autom. Control 1974; 19:716–723.
